# Stromal TGF-β signaling induces AR activation in prostate cancer

**DOI:** 10.18632/oncotarget.2536

**Published:** 2014-10-14

**Authors:** Feng Yang, Yizhen Chen, Tao Shen, Dan Guo, Olga Dakhova, Michael M. Ittmann, Chad J. Creighton, Yiqun Zhang, Truong D. Dang, David R. Rowley

**Affiliations:** ^1^ Department of Molecular and Cellular Biology, Baylor College of Medicine, Houston, TX 77030; ^2^ Pathology and Immunology, Baylor College of Medicine, Houston, TX 77030; ^3^ Medicine, Baylor College of Medicine, Houston, TX 77030

**Keywords:** TGF-β, AR, tumor microenvironment, prostate stroma, prostate cancer, co-culture

## Abstract

AR signaling is essential for the growth and survival of prostate cancer (PCa), including most of the lethal castration-resistant PCa (CRPC). We previously reported that TGF-β signaling in prostate stroma promotes prostate tumor angiogenesis and growth. By using a PCa/stroma co-culture model, here we show that stromal TGF-β signaling induces comprehensive morphology changes of PCa LNCaP cells. Furthermore, it induces AR activation in LNCaP cells in the absence of significant levels of androgen, as evidenced by induction of several AR target genes including PSA, TMPRSS2, and KLK4. SD-208, a TGF-β receptor 1 specific inhibitor, blocks this TGF-β induced biology. Importantly, stromal TGF-β signaling together with DHT induce robust activation of AR. MDV3100 effectively blocks DHT-induced, but not stromal TGF-β signaling induced AR activation in LNCaP cells, indicating that stromal TGF-β signaling induces both ligand-dependent and ligand-independent AR activation in PCa. TGF-β induces the expression of several growth factors and cytokines in prostate stromal cells, including IL-6, and BMP-6. Interestingly, BMP-6 and IL-6 together induces robust AR activation in these co-cultures, and neutralizing antibodies against BMP-6 and IL-6 attenuate this action. Altogether, our study strongly suggests tumor stromal microenvironment induced AR activation as a direct mechanism of CRPC.

## INTRODUCTION

Prostate cancer (PCa) is the most commonly diagnosed cancer and the second leading cause of cancer death in American men. AR signaling is essentially required for most human prostate cancers. Therefore, ADT is the standard treatment for advanced and metastatic PCa. Most prostate tumors initially respond to ADT therapy. However, most tumors will relapse and become the castration-resistant PCa (CRPC). Currently, there is no cure for CRPC. Interestingly, AR signaling is re-activated in most CRPC tumors in the presence of castration level of androgen ligands. Hence, AR remains as a major therapeutic target for CRPC.

Several molecular mechanisms may count for this AR re-activation in CRPC, including AR gene amplification/overexpression, AR mutation, the presence of AR splice variants, enhanced AR co-regulators signaling, alterations in steroid metabolism, growth factor and/or cytokine induced AR activation *etc* [1]. Tumor microenvironment also plays critical roles in regulating prostate cancer progression [2]. Prostate cancer is enriched in reactive stromal microenvironment, including reactive myofibroblasts that are uniquely presented in wound repair and tumor microenvironment [3–5]. Transforming Growth Factor β (TGF-β) is generally overexpressed in most carcinomas associated with a reactive stroma, including breast, colon, and prostate [3, 6–9]. Overexpression of TGF-β in carcinoma cells is usually associated with a down-regulation of functional TGF-β receptors in carcinoma cells but not in stromal cells [9–12]. Subcutaneous injection of TGF-β1 is sufficient to induce a stromal reaction with differentiation to myofibroblasts, enhanced collagen production and stimulated angiogenesis [13, 14]. Therefore, TGF-β1 may be a key factor inducing a reactive stroma in wound repair and cancer. By using the differential reactive stroma (DRS) xenograft model [15–18], we have shown that human prostate stromal cells differentially promote rate of PCa progression [15]. By conditional knockout of TGF-β Receptor II (TβRII) and overexpression of a dominant negative Smad3 in prostate stromal cells in LNCaP DRS xenograft model, we have demonstrated that Smad3-mediated TGF-β signaling in prostate stroma promotes prostate tumor growth and angiogenesis [16, 18] and this stromal TGF-β action is partially mediated by Connective Tissue Growth Factor (CTGF) and Fibroblast Growth Factor 2 (FGF-2) signaling [17, 18]. Therefore, TGF-β signaling in prostate stroma regulates PCa progression.

Interleukin-6 (IL-6) is a pleiotropic cytokine that play important roles in regulating immune system and inflammation. It is also a key cytokine in regulating human cancers, including PCa [19]. Serum IL-6 level is associated with PCa progression and metastasis [20, 21]. Functionally, IL-6 can induce AR expression and AR activation, and promote PCa cell growth [22–28]. IL-6 has also been shown to promote castration-resistance of PCa including that to enzalutamide (MDV3100, a second-generation antiandrogen) [25, 28, 29]. Interestingly, the circulating levels of both IL-6 and TGF-β1 were elevated in patients with metastatic PCa [30]. Bone morphogenetic proteins (BMPs) play important roles in inducing bone formation. BMP-6 expression is frequently elevated in PCa [31]. It can promote PCa bone metastases and its expression is associated with a more invasive phenotype [32, 33]. Interestingly, two most recent studies revealed a role of BMP-6 in promoting castration-resistance of PCa [34, 35].

In this report, we used direct co-cultures of LNCaP cells with three different human prostate stromal cell lines to show that prostate stroma-specific TGF-β signaling induces AR activation in LNCaP cells in the absence of significant amount of androgens, and that treatment of MDV3100, a second-generation antiandrogen [36], only partially attenuates this AR activation. Our study also revealed robust cooperative activity between stromal TGF-β signaling and DHT ligand in inducing AR activation in PCa cells. Finally, we showed that IL-6 and BMP-6 together induces robust AR activation, and that they partially mediate this prostate stromal TGF-β signaling induced AR activation in PCa cells.

## RESULTS

### Prostate stroma – specific TGF-β signaling induces morphological changes in LNCaP cells

**Figure 1:**
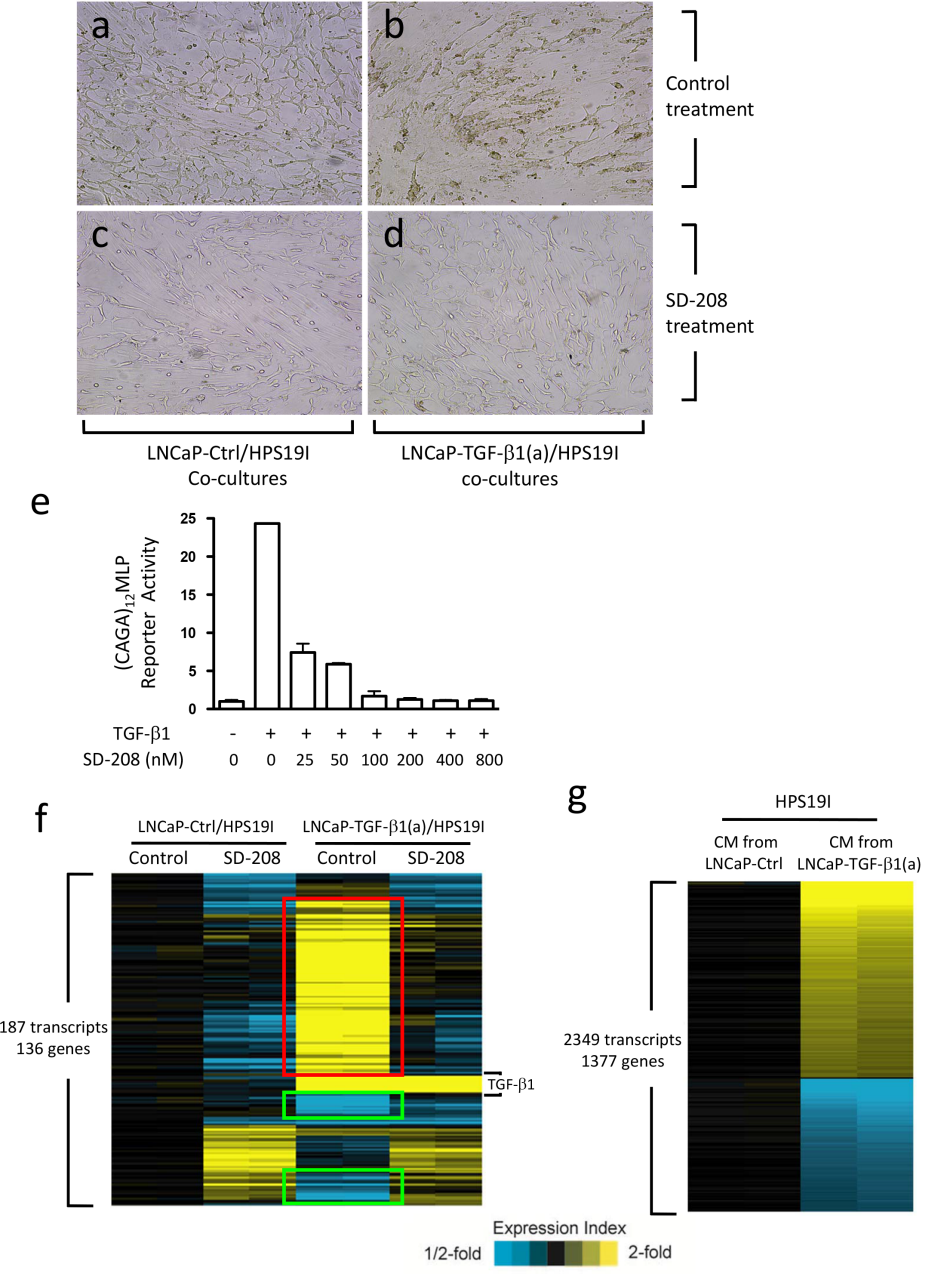
Prostate stromal TGF-β signaling induces profound changes in the co-cultured LNCaP cells **(a-d)** LNCaP-TGF-β1(a) cells and LNCaP-Ctrl cells were co-cultured with HPS19I human prostate stromal cells in RPMI1640 supplemented with 0.2% FBS, and treated with 400 nM of SD-208 or control for 15 days. Representative photographs were shown for **(a)** control and **(c)** SD208 treated LNCaP-Ctrl/HPS19I co-cultures, and **(b)** control and **(d)** SD208 treated LNCaP-TGF-β1(a)/HPS19I co-cultures. HPS19I cells are at the bottom layer. **(e)** HPS19I cells were co-transfected with (CAGA)_12_MLP and pRL-null vectors, and treated with 50 pM of TGF-β1 and different dosages of SD-208 compound for 24 hours. Cell lysates were prepared and assayed for luciferase activity. **(f)** LNCaP-TGF-β1(a) cells or LNCaP-Ctrl cells were co-cultured with HPS19I cells in RPMI1640 supplemented with 0.2% FBS and treated with 400 nM of SD-208 or vehicle control for 6 days. Total RNA was extracted and microarrays were performed to compare differential gene expression among these differentially treated co-cultures. Two independent experiments were performed. **(g)** HPS19I cells were treated for 6 days with conditioned media (CM) from LNCaP-TGF-β1(a) cells or LNCaP-Ctrl cells in RPMI1640 supplemented with 0.2% FBS. Microarrays were performed to compare differential gene expression between these two differentially treated groups. Two independent experiments were performed.

We have previously shown that stromal TGF-β signaling promotes prostate tumor growth [18]. To further delineate the underlying mechanisms, we generated LNCaP cells overexpressing an HA-tagged constitutively activated TGF-β1 ligand (LNCaP-TGF-β1(a)) and control LNCaP cells (LNCaP-Ctrl) as described before [37]. We then performed *in vitro* PCa/stroma co-cultures by plating LNCaP-TGF-β1(a) cells or LNCaP-Ctrl cells on top of the mostly confluent HPS-19I cells, a previously generated human prostate stromal cell line [38]. Since LNCaP cells are defective in TGF-β receptor I (TβRI/ALK-5) that is essential for mediating TGF-β signaling [39], only HPS19I cells can respond to TGF-β ligand in these co-cultures. This provides a unique opportunity to study how prostate stromal cell-specific TGF-β signaling regulates PCa biology. We performed these co-cultures in 0.2% FBS containing minimal amount of growth factors and cytokines for up to 28 days. We found that while the LNCaP-Ctrl cells remained relatively flat, LNCaP-TGF-β1(a) cells formed sphere-like structures in these co-cultures (Figure 1a & b, Supplementary Figure 1), indicating that prostate stromal cell-specific TGF-β signaling induces profound biological changes in LNCaP cells.

SD-208 is a TβRI (ALK-5) protein kinase-specific inhibitor [40]. To determine its efficacy in inhibiting TGF-β signaling in HPS19I cells, we transfected HPS19I cells with a (CAGA)_12_MLP (Smad binding sequence) reporter construct [41] and a pRL-null vector (Promega, control for transfection efficiency). We then treated these cells with 50 pM of TGF-β1 along with different dosages of SD-208. TGF-β1 induced a 25-fold increase in (CAGA)_12_MLP reporter activity in the transfected HPS19I cells, and this induction was inhibited by SD-208 compound in a dose-dependent manner (Figure 1e). 400 nM of SD-208 abolished TGF-β signaling in HPS19I cells, thus was used in the co-culture experiments (Figure 1c and 1d). Consistent with its TGF-β signaling inhibition activity, 400 nM of SD-208 treatment blocked the LNCaP-TGF-β1(a) cells from forming sphere-like structures in the LNCaP-TGF-β1(a)/HPS19I co-cultures (Figure 1d).

### TGF-β induces profound gene expression changes in LNCaP/HPS19I co-cultures

To explore how prostate stroma-specific TGF-β signaling regulates LNCaP cell biology in these co-cultures, we carried out cDNA microarray study to compare differential gene expression among (1) control treated LNCaP-Ctrl/HPS19I co-cultures, (2) 400 nM of SD-208 treated LNCaP-Ctrl/HPS19I co-cultures, (3) control treated LNCaP-TGF-β1(a)/HPS19I co-cultures, and (4) 400 nM of SD-208 treated LNCaP-TGF-β1(a)/HPS19I co-cultures. After 6-day treatment, total RNAs were directly extracted from these co-cultures from two independent studies, reverse transcribed, and submitted for microarray analysis using the 4×44K Whole Human Genome Oligo Microarray chip (Agilent Technologies). We found that 187 transcripts representing 136 unique genes were differentially expressed among these groups (ANOVA P<0.001, standard deviation > 0.2), including 79 unique genes (in red) that were induced by TGF-β, but blocked or reversed by SD-208 treatment, as well as 23 genes (in green) that were repressed by TGF-β, but blocked or reversed by SD-208 treatment (Figure 1f). The most significantly upregulated genes in these LNCaP-TGF-β1(a)/HPS19I co-cultures include TIMP3, COMP, FN1, TSPAN2, CILP, TNFAIP6, ENC1, CDKN2B, MRAS, LTBP2, LOX, POSTN, LRRC32 *etc.*, as well as notably KLK3 (PSA), a prostate epithelial cell specific marker and an AR target gene (Table 1a). The most significantly downregulated genes include UGT2B17, FAM111A, ZNF294, ANK3, MAPRE2 *etc.* (Table 1b). Control and SD-208 treated LNCaP-TGF-β1(a)/HPS19I co-cultures all exhibited overexpression of nine transcripts, all of which represent TGF-β1, the ectopically overexpressed gene in LNCaP-TGF-β1(a) cells.

**Table 1A:** Most up-regulated genes in LNCaP-TGF-β1(a)/HPS19I co-cultures (microarray)

RefSeq Nuc	Gene	TGF-β1+Ctrl vs. Ctrl+Ctrl	TGF-β1+SD208 vs.TGF-β1+Ctrl	p-value (ANOVA)
NM_000362	TIMP3	21.80112275	0.035626662	0.000969463
NM_000095	COMP	18.43130951	0.063331859	3.72E-05
NM_212482	FN1	11.94239303	0.12931702	0.000623914
NM_005725	TSPAN2	10.28542467	0.101405721	9.73E-05
NM_003613	CILP	10.21670312	0.091015909	0.000805663
NM_007115	TNFAIP6	8.549476011	0.117397514	1.33E-06
NM_003633	ENC1	8.46863116	0.132486872	0.000143214
NM_078487	CDKN2B	7.857379819	0.142359863	0.000247918
NM_012219	MRAS	6.906686977	0.120416016	3.32E-05
NM_000428	LTBP2	6.530185903	0.17879509	0.00021206
NM_002317	LOX	6.253405306	0.162652276	0.000493453
NM_006475	POSTN	6.131065054	0.268041215	0.000381746
NM_005512	LRRC32	6.027300939	0.155609222	0.000244534
NM_001013398	IGFBP3	5.892165506	0.181799757	0.000340864
NM_000660	TGFB1	5.448264587	0.78251392	0.000170351
NM_002667	PLN	5.105920007	0.214734252	0.000696765
NM_014632	MICAL2	4.972581483	0.17152412	0.000559198
NM_016931	NOX4	4.849811685	0.225379795	0.000265966
NM_014631	SH3PXD2A	4.341337763	0.24444475	0.000103435
NM_000393	COL5A2	4.280097929	0.165513816	5.77E-05
NM_003474	ADAM12	4.189365259	0.274394503	0.000101548
NM_014467	SRPX2	4.147952958	0.257998723	4.06E-05
NM_006851	GLIPR1	4.092470638	0.201018413	4.62E-05
NM_005613	RGS4	4.074850693	0.251673169	4.92E-05
NM_001018004	TPM1	3.947191935	0.195719983	0.000839361
NM_006329	FBLN5	3.701593985	0.245752501	0.000232936
BC014203	FAM101B	3.623415448	0.309548366	0.000792052
NM_000399	EGR2	3.490980445	0.261212761	0.00023454
NM_001648	KLK3	3.481810093	0.288453944	0.000272881
NM_000096	CP	3.324909574	0.332527772	0.000485262
NM_001898	CST1	3.287001556	0.335459083	0.00068421
NM_181847	AMIGO2	3.249577889	0.261229048	0.000858199
NM_138455	CTHRC1	3.217756975	0.313332784	0.000253826
NM_153026	PRICKLE1	3.179655624	0.38858927	0.000640482
NM_012293	PXDN	3.039816136	0.257492324	0.000777339

**Table 1B:** Most down-regulated genes in LNCaP-TGF-β1(a)/HPS19I co-cultures (microarray)

RefSeq Nuc	Gene	TGF-β1+Ctrl vs. Ctrl+Ctrl	TGF-β1+SD208 vs.TGF-β1+Ctrl	p-value (ANOVA)
NM_001077	UGT2B17	0.196197102	4.554852029	0.000695519
NM_022074	FAM111A	0.33585709	2.325687499	0.000978972
NM_015565	ZNF294	0.356016518	2.631507725	0.000769972
AK126851	ANK3	0.413492187	3.394052268	9.89916E-05
NM_014268	MAPRE2	0.447337037	2.56792461	0.000371084
NM_002467	MYC	0.484325292	2.653538875	0.000233651
NM_003759	SLC4A4	0.489633359	0.985494206	0.00077358
NM_021190	PTBP2	0.490358498	1.585425699	0.000671709
AK075235	SVEP1	0.521242168	1.139842928	5.82E-04
NM_018689	KIAA1199	0.550188967	1.452227923	6.60136E-05
NM_000214	JAG1	0.572787648	1.261023084	0.000639491
NM_001634	AMD1	0.633893667	1.61911846	9.31E-05
NM_006358	SLC25A17	0.651353495	1.882434831	0.000460944
NM_002113	CFHR1	0.672707916	1.258311297	7.55E-04
NM_017686	GDAP2	0.692205019	1.111019264	0.000774186
NM_058191	C21orf66	0.694078519	1.479905697	7.82E-05
NM_004687	MTMR4	0.697685088	1.504510216	0.000276199

In order to identify the prostate epithelia-specific gene that was regulated by prostate stromal TGF-β signaling, we also treated HPS19I cells alone using conditioned media collected from LNCaP-TGF-β1(a) cells or LNCaP-Ctrl cells cultured in RPMI1640 supplemented with 0.2% FBS. Conditional media, but not TGF-β1 ligand, were used here to more closely simulate the co-culture conditions. After 6 days of treatment, we extracted total RNA from these HPS19I cells and performed microarray. 2349 transcripts representing 1377 genes were differentially expressed among these two treatment groups (fold change>1.5, Figure 1g). The most significantly upregulated genes in HPS19I cells include ELN, EGR2, COMP, NOX4, CILP, CDKN2B, ENC1, AGT, PMEPA1, TNFAIP6, TIMP3 *etc.* (Table 2a), and most significantly downregulated genes include ADH1C, ADH1A, ALDH1A1, VCAM1, HSD17β2, SMPDL3A, ZFP36L2, SECTM1, ADAMTS5, NR2F1 *etc*. (Table 2b).

**Table 2A:** Most up-regulated genes in LNCaP-TGF-β1(a) cell conditioned media treated HPS19I cells (microarray)

RefSeq Nuc	Gene	TGF-β1 CM vs. Ctrl CM
BC065566	ELN	16.67449355
NM_000399	EGR2	14.05299758
NM_000095	COMP	12.98900057
NM_016931	NOX4	12.32605115
NM_003613	CILP	9.059147489
NM_078487	CDKN2B	8.803843969
NM_003633	ENC1	8.260572524
NM_000029	AGT	7.191604093
NM_020182	PMEPA1	7.070540703
NM_007115	TNFAIP6	6.750100868
X77690	TIMP3	6.366782695
NM_004385	VCAN	6.149750603
NM_012219	MRAS	5.992620419
NM_000501	ELN	5.807745301
NM_006216	SERPINE2	5.755856917
NM_005725	TSPAN2	5.661870275
NM_005725	TSPAN2	5.352542656
NM_031866	FZD8	5.312916891
NM_002318	LOXL2	5.290455686
NM_020152	C21orf7	5.266205737
NM_002667	PLN	4.972344608
NM_054034	FN1	4.877547028
NM_014631	SH3PXD2A	4.589092327
NM_014399	TSPAN13	4.524283014
NM_003474	ADAM12	4.349125589
NM_182943	PLOD2	4.30726254
NM_014632	MICAL2	4.05405353
NM_001013398	IGFBP3	4.052272457
NM_006475	POSTN	4.052068264
NM_000846	GSTA2	4.041328015
NM_000428	LTBP2	4.023595863
NM_002317	LOX	3.946875107
NM_004750	CRLF1	3.89952373
NM_012261	C20orf103	3.871335965
NM_014840	NUAK1	3.628631621
NM_005613	RGS4	3.583530694
NM_001845	COL4A1	3.569510901
NM_138409	MRAP2	3.500158747

**Table 2B:** Most down-regulated genes in LNCaP-TGF-β1(a) cell conditioned media treated HPS19I cells (microarray)

RefSeq Nuc	Gene	TGF-β1 CM vs. Ctrl CM
NM_000669	ADH1C	0.048337642
NM_000667	ADH1A	0.049204652
NM_018689	KIAA1199	0.055600352
NM_000689	ALDH1A1	0.090425225
NM_001078	VCAM1	0.100168911
NM_002153	HSD17B2	0.123017582
NM_006714	SMPDL3A	0.124668133
NM_006887	ZFP36L2	0.1669457
NM_003004	SECTM1	0.181835508
NM_007038	ADAMTS5	0.184125165
NM_007021	C10orf10	0.184605811
NM_005654	NR2F1	0.206777199
NM_004753	DHRS3	0.207888106
NM_014585	SLC40A1	0.208805616
NM_012342	BAMBI	0.21522776
NM_000891	KCNJ2	0.218105292
NM_001031716	OBFC2A	0.222516674
NM_019105	TNXB	0.22609769
NM_020379	MAN1C1	0.228067582
NM_005329	HAS3	0.228406025
NM_001430	EPAS1	0.232550842
NM_030781	COLEC12	0.239762468
NM_005602	CLDN11	0.239772493
NM_005410	SEPP1	0.245152972
NM_006287	TFPI	0.246145446
NM_018043	ANO1	0.247547036
NM_000076	CDKN1C	0.247573166
NM_001083	PDE5A	0.248350261
NM_018043	ANO1	0.250724637
NM_198194	STOM	0.251440257
NM_002825	PTN	0.255859758
NM_153225	C8orf84	0.256217479
NM_018487	TMEM176A	0.258119323
NM_001146	ANGPT1	0.25867152
NM_000599	IGFBP5	0.261560301
NM_014020	TMEM176B	0.262431295
NM_175861	TMTC1	0.270304699
NM_006988	ADAMTS1	0.275587107

We compared the obtained TGF-β regulated gene lists in the LNCaP/HPS19I co-cultures and those in the HSP19I cells cultured alone. As expected, most of the TGF-β regulated genes in the LNCaP/HPS19I co-cultures were also presented in the TGF-β treated HSP19I cells, indicating that these genes might also be TGF-β regulated genes in HPS19I cells of these co-cultures. Again, PSA (KLK3), a prostate epithelial cell specific and AR regulated gene, was induced in the LNCaP-TGF-β1 (a) / HPS19I cell co-cultures, but not expressed in HPS19I cells when cultured alone. Furthermore, SD-208 treatment blocked this induction (Table 1a).

### Prostate stromal TGF-β signaling induces AR activation in PCa cells

**Figure 2:**
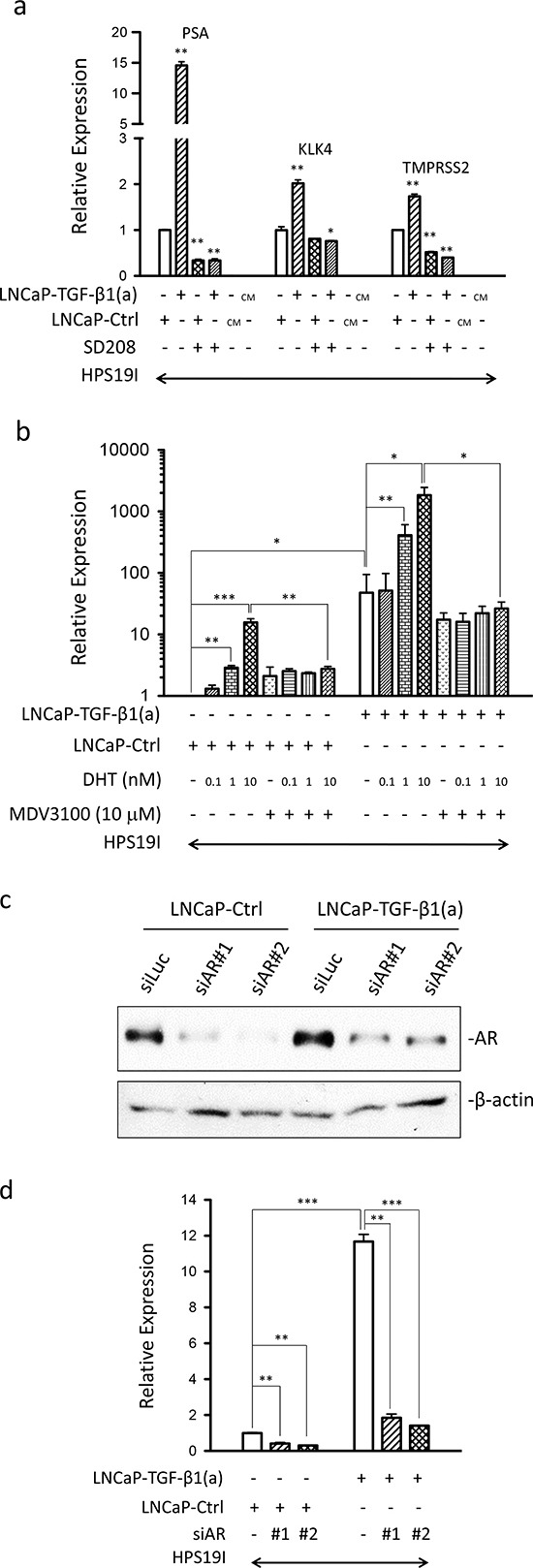
Prostate stromal TGF-β signaling induces AR activation in the co-cultured LNCaP cells **(a)** qPCR were used to analyze the expression of PSA, KLK4, and TMPRSS2 in the differentially treated LNCaP-TGF-β1(a)/HPS19I, LNCaP-Ctrl/HPS19I, and HPS19I cells as described in Figure 1f & g. CM: conditioned media. Representative data is from two independent experiments. Gene expression data were normalized to GAPDH expression. *p<0.05, **p<0.01 (unpaired Student's t-test). **(b)** LNCaP-TGF- β1(a) cells and LNCaP-Ctrl cells were co-cultured with HPS19I cells in RPMI1640 supplemented with 0.2% charcoal-stripped FBS, and treated with different dosages of DHT in the presence (+) or absence (−) of 10 μM of MDV3100 for 6 days. Total RNA was extracted and reverse transcribed. qPCR were used to analyze the expression of PSA, KLK4, and TMPRSS2 in these co-cultures. Data is from three independent experiments. Gene expression data were normalized to GAPDH expression. *p<0.05, **p<0.01, ***p<0.001 (paired Student's t-test). (**c, d**) LNCaP-TGF-β1(a) cells and LNCaP-Ctrl cells were transfected with two independent siRNAs against human AR (siAR#1 and siAR#2), along with siRNA against luciferase (siLuc) as control. On the second day, the cells were trypsinized and collected. (**c**) Part of the cells were used for continuing culturing in regular media for 4 days, after which cell lysates were prepared and subjected to Western blot for AR and β-actin. (**d**) Part of the cells were used for co-cultures with HPS19I cells in RPMI1640 supplemented with 0.2% charcoal-stripped FBS for 4 days, after which total RNA was extracted and reverse transcribed. qPCR were used to analyze the expression of PSA in these co-cultures. Representative data is from two independent experiments. Gene expression data were normalized to GAPDH expression. **p<0.01, ***p<0.001 (unpaired Student's t-test).

Since PSA is a well-established AR target gene in human PCa, our above observation suggested that stroma-specific TGF-β signaling might induce AR activation in PCa cells. To test this, we performed qPCR on the expression of additional well-established PCa epithelia-specific AR target genes including KLK4 and TMPRSS2 in these LNCaP/HPS19I co-cultures. TGF-β induced the expression of all these three AR target genes in the LNCaP-TGF-β1(a)/HPS19I co-cultures, but not in LNCaP cells (data not shown) or HPS19I cells cultured alone (Figure 2a). Furthermore, these inductions were blocked by addition of 400 nM of SD-208, further confirming that TβRI (ALK5) protein kinase activity in the HPS19I cells was required for TGF-β ligand induced AR activation in LNCaP cells (Figure 2a).

To further reduce androgen levels, we also performed LNCaP-TGF-β1(a)/HPS19I co-cultures and LNCaP-Ctrl/HPS19I co-cultures in RPMI1640 media supplemented with 0.2% charcoal stripped FBS deprived of androgen, and treated them with increasing dosages of DHT and/or 10 μM of MDV3100, a second-generation antiandrogen [36], for six days. We similarly extracted total RNA directly from these co-cultures, and performed reverse transcription reactions and qPCR analysis for PSA expression. As expected, DHT induced PSA expression in the control LNCaP-Ctrl/HPS19I co-cultures in a dose-dependent manner. We also unexpectedly observed that MDV3100 treatment somehow modestly induced PSA expression in these LNCaP-Ctrl/HPS19I co-cultures maintained in 0.2% charcoal stripped FBS, whereas the AR antagonist activity of MDV3100 was confirmed by its strong inhibition of 10 nM DHT induced PSA expression in these LNCaP-Ctrl/HPS19I co-cultures (Figure 2b). Consistent with the data obtained in LNCaP/HPS19I co-cultures in 0.2% FBS (Figure 2a), PSA expression in the LNCaP-TGF-β1(a)/HPS19I co-cultures in 0.2% charcoal-stripped FBS was also greatly induced, to levels comparable to 10nM DHT induced PSA expression in LNCaP-Ctrl/HPS19I co-cultures. These observations further confirmed prostate stroma-specific TGF-β signaling induction of AR activation in the absence of significant amount of androgen. Addition of DHT dose-dependently promoted PSA expression in these LNCaP-TGF-β1(a)/HPS19I co-cultures, with robust induction observed in 1 nM and 10 nM of DHT treatments, indicating strong cooperative or additive actions between the stromal TGF-β signaling induced AR activation and the DHT ligand-induced AR activation. Consistent with the AR antagonist activity, 10 μM of MDV3100 robustly blocked 1nM and 10 nM DHT-induced PSA expression in the LNCaP-TGF-β1(a)/HPS19I co-cultures. In contrast, MDV3100 treatment only modestly reduced stromal TGF-β signaling induced PSA expression in the absence of externally added DHT ligand, indicating that a large fraction of stromal TGF-β signaling induced AR activation is ligand-independent (Figure 2b). Finally, LNCaP-TGF-β1(a) cells and LNCaP-Ctrl cells expressed comparable levels of basal and DHT-induced PSA (Supplementary Figure 2), further confirming that stroma-specific TGF-β signaling is essential for inducing AR activation in LNCaP cells in these co-cultures.

To confirm that our observed stromal TGF-β signaling induced PSA expression is AR dependent, we performed knockdown of AR in LNCaP-Ctrl and LNCaP-TGF-β1(a) cells using two independent siRNAs against human AR. Western blots confirmed significant knockdown of AR in both cell lines (Figure 2c). These AR-knockdown LNCaP cells were used for co-cultures with HPS19I cells in RPMI1640 media supplemented with 0.2% charcoal stripped FBS for four days. We extracted total RNA directly from these co-cultures, and performed reverse transcription reactions and qPCR analysis for PSA expression. Indeed, knockdown of AR in LNCaP cells greatly inhibited PSA expression in these co-cultures, confirming that stromal TGF-β signaling induced PSA expression is dependent on AR (Figure 2d).

**Figure 3:**
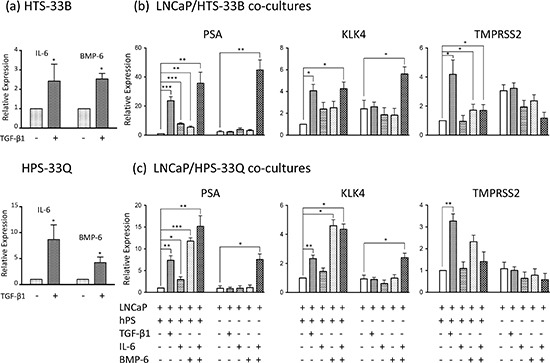
IL-6 and BMP-6 induces AR activation in LNCaP cells **(a)** HTS33B and HPS33Q human prostate stromal cells were cultured in RPMI1640 supplemented with 0.2% charcoal-stripped FBS and treated with TGF-β1 (50 pM) for 3 days. Total RNA was extracted and reverse transcribed. qPCR were used to analyze the expression of IL-6 and BMP-6. Data is from three independent experiments. **(b)** LNCaP cells were co-cultured with HTS33B cells in RPMI1640 supplemented with 0.2% charcoal-stripped FBS, and treated with TGF-β1 (50 pM), IL-6 (25 ng/ml), and/or BMP-6 (50 ng/ml) for 6 days. Total RNA was extracted and reverse transcribed. qPCR were used to analyze the expression of PSA, KLK4, TMPRSS2 expresssion in these co-cultures. Data is from three independent experiments. **(c)** LNCaP cells were similarly co-cultured with HPS33Q cells and treated with TGF-β1 (50 pM), IL-6 (25 ng/ml), and/or BMP-6 (50 ng/ml) for 6 days, and analyzed for the expression of PSA, KLK4, TMPRSS2 (qPCR). Data is from three independent experiments. All gene expression data were normalized to GAPDH expression. hPS (human prostate stromal cells) stands for either HTS33B **(b)** or HPS33Q cells **(c)**. *p<0.05, **p<0.01, ***p<0.001 (paired Student's t-test).

To further expand our observation, we also performed co-cultures of LNCaP cells with HTS33B cells and HPS33Q cells, two additional human prostate stromal cell lines that we prepared following a previously described protocol [5, 38]. These LNCaP/HTS33B co-cultures and LNCaP/HPS33Q co-cultures were treated with 50 pM of TGF-β1 or vehicle control in RMPI1640 supplemented with 0.2% charcoal stripped FBS for six days. Total RNA was similarly directly extracted from these co-cultures, reverse transcribed, and used for qPCR analysis for gene expression. We showed that TGF-β treatment induced the expression of PSA, KLK4, and TMPRSS2 in all these co-cultures, further confirming that prostate stromal cell-specific TGF-β signaling induces AR activation in LNCaP cells (Figure 3b, c).

### IL-6 and BMP-6 activity partially mediates prostate stromal TGF-β signaling induced AR activation in LNCaP cells

IL-6 is a key cytokine regulating PCa biology, including inducing AR activity and promoting androgen-independent growth [22–25]. BMP-6 was recently reported to promote castration-resistant PCa [34, 35]. We found that TGF-β induces the expression of both IL-6 and BMP-6 in human prostate stromal cells cultured in 0.2% charcoal stripped FBS (Figure 3a). To examine whether IL-6 and/or BMP-6 mediate the stromal TGF-β signaling induced AR activation in LNCaP cells, we first treated LNCaP/HTS33B co-cultures and LNCaP/HPS33Q co-cultures in RMPI1640 supplemented with 0.2% charcoal stripped FBS with 25 ng/ml of IL-6 and/or 50 ng/ml of BMP-6 for six days. While IL-6 treatment and BMP-6 treatment each can induce PSA expression in all these co-cultures, co-treatments of both IL-6 and BMP-6 robustly induced PSA expression to levels exceeding those induced by TGF-β treatment. These results indicate that IL-6 and BMP-6 together strongly induces AR activation in LNCaP cells and that this action may greatly contribute to stromal TGF-β signaling induced AR activation in LNCaP cells.

We next examined whether the presence of prostate stromal cells is required for the IL-6/BMP-6 induced AR activation in LNCaP cells. In contrast to our above observations in the co-culture system, neither IL-6 treatment alone nor BMP-6 treatment alone can greatly enhance PSA expression in LNCaP cells cultured alone in RPMI 1640 media supplemented with 0.2% charcoal stripped FBS. However, co-treatments of both IL-6 and BMP-6 induced robust expression of PSA in the LNCaP-only cell cultures, further confirming the cooperative activities of IL-6 and BMP-6 in inducing AR activation in LNCaP cells (Figure 3b, c). We made similar observations in KLK4 expression, but not in TMPRSS2 expression, in these IL-6 and BMP-6 treated LNCaP/stromal co-cultures as well as in LNCaP cells cultured alone (Figure 3b, c). These data suggest that although IL-6 and BMP-6 may greatly contribute to stromal TGF-β signaling induced AR activities including inducing PSA and KLK4 expression, additional signaling molecules/pathways may be required for mediating stromal TGF-β signaling induced other AR activities such as inducing TMPRSS2 expression.

**Figure 4:**
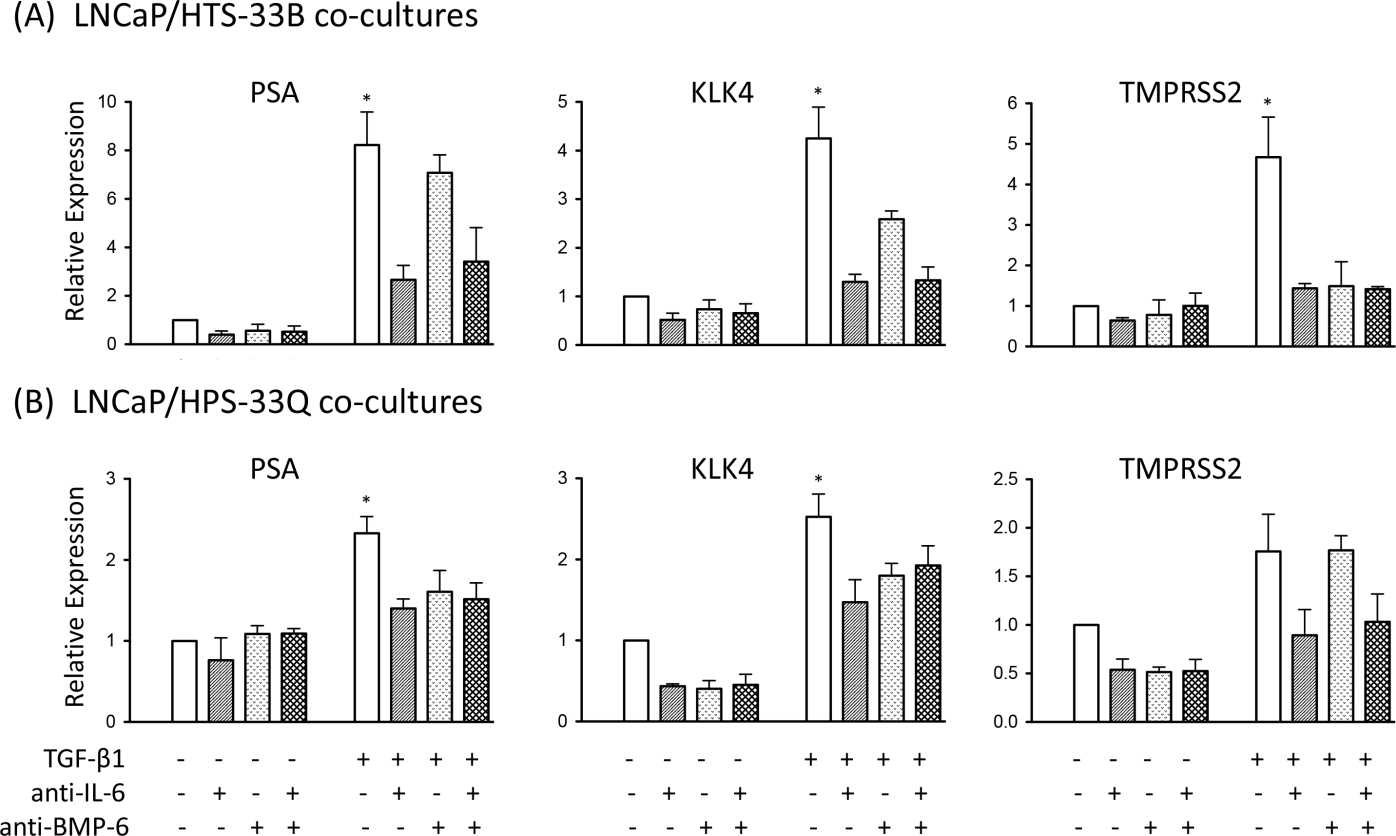
IL-6 and BMP-6 neutralizing antibody treatment attenuates stromal TGF-β induced AR activation in the co-cultured LNCaP cells **(a)** LNCaP cells were cultured alone or co-cultured with HTS33B cells in RPMI1640 supplemented with 0.2% charcoal-stripped FBS, and treated with TGF-β1 (50 pM) along with neutralizing antibody against IL-6 and/or BMP-6 (both at 2 μg/ml) for 6 days. Total RNA was extracted and reverse transcribed. qPCR were used to analyze the expression of PSA, KLK4, TMPRSS2 expresssion in these co-cultures. Data is from two independent experiments. **(b)** LNCaP cells were similarly cultured alone or co-cultured with HPS33Q cells and treated with TGF-β1 (50 pM) along with neutralizing antibody against IL-6 and/or BMP-6 (both at 2 μg/ml) for 6 days, and analyzed for the expression of PSA, KLK4, TMPRSS2 (qPCR). Data is from two independent experiments. All gene expression data were normalized to GAPDH expression. *p<0.05 (unpaired Student's t-test).

Finally, to further evaluate the functional roles of IL-6 and BMP-6 in mediating stromal TGF-β signaling induced AR activation in LNCaP cells, we treated the LNCaP/HTS33B and LNCaP/HPS33Q co-cultures with IL-6 and BMP-6 neutralizing antibodies. Use of IL-6 neutralizing antibody produced an apparent reduction in stromal TGF-β signaling induced AR activation in LNCaP cells, whereas BMP-6 neutralizing antibody attenuated this induction to a less extent (Figure 4a, b). Altogether, these data suggest that IL-6 and BMP-6 mediate prostate stromal TGF-β signaling induced AR activation.

## DISCUSSION

Tumor microenvironment is important for prostate cancer progression. TGF-β is a key cytokine in regulating cancer progression, either by directly regulating tumor cells, such as inducing epithelial to mesenchymal transition (EMT), or indirectly by regulating tumor microenvironment, such as inducing extracellular matrix remodeling, regulating angiogenesis as well as immune responses. We showed here that TGF-β could indirectly induce AR activation in PCa cells through directly modulating stromal cells in tumor microenvironment. Since AR re-activation is requisite for the recurrence of most CRPC tumors, this stromal TGF-β signaling induced AR activation in PCa cells may provide a direct mechanism for CRPC. Importantly, stromal TGF-β signaling greatly potentiated and enhanced DHT-induced AR activation, suggesting that it might be a therapeutic target to inhibit AR activation in CRPC tumors in the presence of castration-levels of androgen.

Our current data suggest that IL-6 and BMP-6 might partially mediate stromal TGF-β signaling induced AR activation. Interestingly, although neither IL-6 nor BMP-6 could robustly induce AR activation in LNCaP cells when cultured alone, IL-6 and BMP-6 together greatly enhance AR activity in these cells. Notably, when prostate stromal cells are presented in the LNCaP/stromal cell co-cultures, IL-6 and BMP-6 can each significantly enhance AR activation in LNCaP cells, which may attribute to the basal activation of IL-6 and BMP-6 signaling pathway in these co-cultures. As the expression of IL-6 and BMP-6 are elevated in the tumor microenvironment, these may be important factors in the evolution of castration resistant disease.

Consistent with many previous reports, MDV3100 showed robust AR antagonist activities in most of our LNCaP/HPS19I co-cultures studies. However, MDV3100 also exhibited slight apparent AR agonist activity under certain experimental conditions, such as in LNCaP/HPS19I cell co-cultured in 0.2% of charcoal-stripped FBS as described in Figure 2. The nature and significance of this unexpected slight AR agonist activity of MDV3100 is still to be determined. Finally, stromal TGF-β signaling induced AR activation in LNCaP cells can only be partially attenuated by treatment of MDV3100 (Figure 2), indicating both ligand-dependent and ligand-independent AR activation. Interestingly, our microarray study and qPCR analysis revealed that stromal TGF-β signaling strongly repressed expression of UGT2B17 (Table 1b), as well as UGT2B15 (data not shown), two key epithelium-specific UDP-glucuronosyltransferases for glucuronidation and inactivation of testosterone and DHT ligands for their efficient clearance from normal prostate epithelial cells as well as PCa cells [42, 43]. These suggest a possibility that repression of UGT2B15 and UGT2B17 in PCa cells may contribute to stromal TGF-β signaling induced ligand-dependent AR activation in PCa cells, which warrants future studies on the functional roles of these two UDP-glucuronosyltransferases in regulating prostate stroma induced AR activation in PCa cells.

## MATERIALS AND METHODS

### Reagents

The Porcine TGF-β1(204-B-002), recombinant human BMP-6 (507-BP), recombinant human IL-6 (206-IL-010), antibodies against human BMP-6 (AF-507), human IL-6 (AF-206-NA), and normal goat IgG (AB-108-C) were purchased from R&D System (Minneapolis, MN, USA). MVD3100 was purchased from Selleckchem (Houston, TX, USA). SD-208 was obtained from *Scios, Inc*. (now Alza Corporation Vacaville, CA, USA; part of Johnson and Johnson).

### Cell cultures

LNCaP cells were purchased from ATCC and cultured in RPMI1640 supplemented with 10% FBS (Hyclone, Logan, UT or Invitrogen, Carlsbad, CA, USA). LNCaP-TGF-β1(a) cells overexpressing an HA-tagged constitutively activated TGF-β1 ligand, HA-TGF-β1(a), and control LNCaP (LNCaP-Ctrl) cells were generated as previously described [37]. Human prostate stromal cell lines HPS19I, HTS33B, and HPS33Q were prepared following a previously described protocol and cultured in Bfs medium: DMEM supplemented with 5% FBS (Hyclone or Invitrogen), 5% Nu serum (Collaborative Research, Bedford, MA), 0.5 μg/mL testosterone, 5 μg/mL insulin, 100 units/mL penicillin, and 100 μg/mL streptomycin (Sigma, St. Louis, MO, USA) [5, 38].

### Human prostate cancer cell and prostate stromal cell co-cultures

HPS19I, HTS33B, and HPS33Q cells were first allowed to grow in Bfs media to confluence on 6 well plates. LNCaP cells were then plated on top of the stromal cells at 1×10^5^ cells per well and subjected to various treatments in RPMI1640 supplemented with 0.2% FBS or charcoal stripped FBS for 6 to 28 days. The treatments include 50 pM (1.25ng/ml) porcine TGF-β1 (R&D Systems), 400 nM of SD-208, 0.1, 1, and 10 nM of DHT (Sigma), 10 μM of MDV3100 (Selleckchem, Houston, TX, USA), 25 ng/ml of IL-6, 50 ng/ml of BMP-6, 2 μg/ml of IL-6 neutralizing antibody, 2 μg/ml of IL-6 neutralizing antibody (R&D system), and various vehicle controls. When applicable, LNCaP cells and prostate stromal cells were each similarly cultured and treated as those for the LNCaP/Stroma co-cultures. Total RNA was extracted from these co-cultures using the RNeasy Miniprep kit (Qiagen, Valencia, CA, USA) or TRIzol® reagent (Invitrogen).

### Knockdown of AR in LNCaP cells

Two siRNAs from Invitrogen, AR/HSS179973 (siRNA#1) and AR/HSS179972 (siRNA#2), were used to knock down AR expression in LNCaP-TGF-β1(a) cells and LNCaP-Ctrl cells. The corresponding sequences are CCGGAAGCUGAAGAAACUUGGUAAU (sense), AUUACCAAGUUUCUUCAGCUUCCGG (antisence) for AR/HSS179973, and GAUGAAGCUUCUGGGUGUCACUAUG (sense), CAUAGUGACACCCAGAAGCUUCAUC (antisense) for AR/HSS179972. siRNAs were transfected into LNCaP cells using GenMute™ siRNA Transfection Reagent following manufacture's protocol (SignaGen, Rockville, MD, USA). Western blots were used to verify knockdown of AR. Antibodies used are anti-AR (sc-816, Santa Cruz Biotechnology, Dallas, TX, USA) and anti-β-actin (A1978, Sigma).

### Luciferase reporter assay

HPS19I cells were transfected with 0.4 μg of (CAGA)_12_MLP [41] and 0.1 μg of pRL-null (Promega, Madison, WI, USA) in 12 well plate using FuGENE 6 transfection reagent (Roche Applied Sciences, Penzberg, Germany) as described before [18]. 24 hours later, cells were treated with 50 pM TGF-β1 and different dosages of SD-208 compound for 24 hours. Cell lysates were prepared and measured for luciferase activity using the Dual-Luciferase^®^ Reporter Assay system (Promega). The reporter firefly luciferase activity was normalized to the co-transfected pRL-null Renilla luciferase activity.

### cDNA microarray

HPS19I cells were grown to mostly confluence in T75 flasks. 5×10^5^ of LNCaP-TGF-β1(a) cells or LNCaP-Ctrl cells were then plated on top of the HPS19I cells. The co-cultures were maintained in RPMI1640 supplemented with 0.2% FBS and subjected to treatments with 400 nM of SD-208 or vehicle control for 6 days. Conditioned media from LNCaP-TGF-β1(a) cells and LNCaP-Ctrl cells maintained in RPMI1640 supplemented with 0.2% FBS were also collected and used to treat HPS19I cells for 6 days. Total RNA was extracted from these LNCaP-TGF-β1(a)/HPS19I and LNCaP-Ctrl/HPS19I co-cultures and from the differentially treated HPS19I cells using the RNeasy Miniprep kit (Qiagen). The cDNA reverse transcription and fluorescent labeling reactions were carried out using the SuperScript^®^ Plus Direct cDNA Labeling System with Alexa Fluor^®^ aha-dUTPs kit (Invitrogen). Briefly 3 μg of RNA and 3 μg of Universal Human Reference RNA (Stratagene, La Jolla, CA) were labeled in reverse transcription with Alexa647 and Alexa555 aha-dUTPs respectively for each sample. After labeling, each cDNA was mixed with reference cDNA and purified with SuperScript Plus Direct cDNA Labeling System purification module according to manufacturer's instructions. Eluted sample was mixed with 10xBlocking Solution and 2xHiRPM buffer (Agilent Technologies, Santa Clara, CA, USA), incubated for 2 min at 95–98° C and hybridized on 4×44K Whole Human Genome Oligo Microarray chip using SureHyb DNA Microarray Hybridization Chambers in DNA Microarray hybridization oven (Agilent Technologies) at 10 rpm, 65°C for 20 hrs. After hybridization, slides were washed in Gene Expression Wash Buffer 1 and 2 for 1 min and than dried by centrifugation at 2000 rpm for 2 min. Microarrays were scanned with a dynamic autofocus microarray scanner (Agilent Microarray Scanner- G2565BA, Agilent Technologies,) using Agilent-provided parameters (Red and Green PMT were each set at 100%, and scan resolution was set to 5 um). The Feature Extraction Software v9.1.3.1 (Agilent Technologies) was used to extract and analyze the signals.

The microarray data were processed using bioconductor (loess normalization), with Combat to adjust for batch effects [44]. Fold changes and ANOVA tests for each gene were computed using log-transformed values. Array data have been deposited into the Gene Expression Omnibus (GSE51624).

### Quantitative PCR

1 μg total RNA was reverse transcribed with random hexamer primers using the SuperScript III First Strand Synthesis kit (Invitrogen). Reversed transcribed product was then analyzed by real-time PCR using Platinum SYBR Green qPCR SuperMix-UDG with ROX (Invitrogen) on the ABI Prism PE7700 sequence analyzer (Applied Biosystems, Foster City, CA, USA) or using the PerfeCTa CYBR Green FastMix (Quanta Biosciences, Gaithersburg, MD, USA) on the Light Cycler^®^ 480II (Roche Applied Science). PCR primers used are PSA: 5′ ACCAGAGGAGTTCTTGACCCCAAA 3′ and 5′ CCCCAGAATCACCCGAGCAG 3′, KLK4: 5′ GGCACTGGTCATGGAAAACGA 3′ and 5′ TCAAGACTGTGCAGGCCCAGCC 3′ (Annealing temperature 56), TMPRSS2: 5′ CGCTGGCCTACTCTGGAA 3′ and 5′ CTGAGGAGTCGCACTCTATCC 3′, GAPDH: 5′ AGCCACATCGCTCAGACAC 3′ and 5′ GCCCAATACGACCAAATCC 3′, IL-6: 5′ CAAAGATGGCTGAAAAAGATGGA 3′ and 5′ CTGTTCTGGAGGTACTCTAGGT 3′, and BMP-6: 5′ GGACAGCGCCTTCCTCAAC 3′ and 5′ CGTACTCCACCAGGTTCACAAA 3′. Relative expression levels were determined by the ΔΔCt method, and normalized to GAPDH.

### Statistics

The statistical analyses on microarray data were described above. Statistical analyses (Student's t-tests) for comparing gene expression were generated using Graphpad Prism 5.0 (GraphPad Software, La Jolla, CA, USA). *P* < 0.05 was considered statistically significant.

## SUPPLEMENTARY FIGURES


