# Supplementary material for: Stromal TGF-β signaling induces AR activation in prostate cancer
Source: Oncotarget. 2014 Oct 14;5(21):10854–69. doi: 10.18632/oncotarget.2536 (PMC4279415; doi:10.18632/oncotarget.2536)
Supplement: Supplementary file 1 [file oncotarget-05-10854-s001.pdf]

SUPPLEMENTARY FIGURES

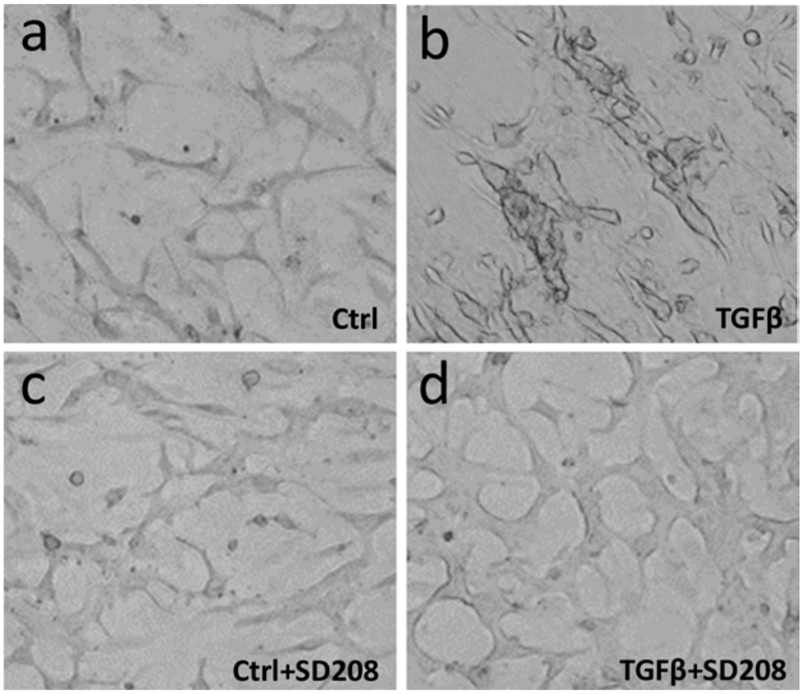

Supplementary Figure 1: Prostate stromal TGF-β signaling induces profound changes in the co-cultured LNCaP cells (higher magnification). (a-d) LNCaP-TGF-β1(a) cells and LNCaP-Ctrl cells were co-cultured with HPS19I human prostate stromal cells in RPMI1640 supplemented with 0.2% FBS, and treated with 400 nM of SD-208 or control for 14 days. Representative photographs were shown for **(a)** control and **(c)** SD208 treated LNCaP-Ctrl/HPS19I co-cultures, and **(b)** control and **(d)** SD208 treated LNCaP-TGF-β1(a)/HPS19I co-cultures. HPS19I cells are at the bottom layer.

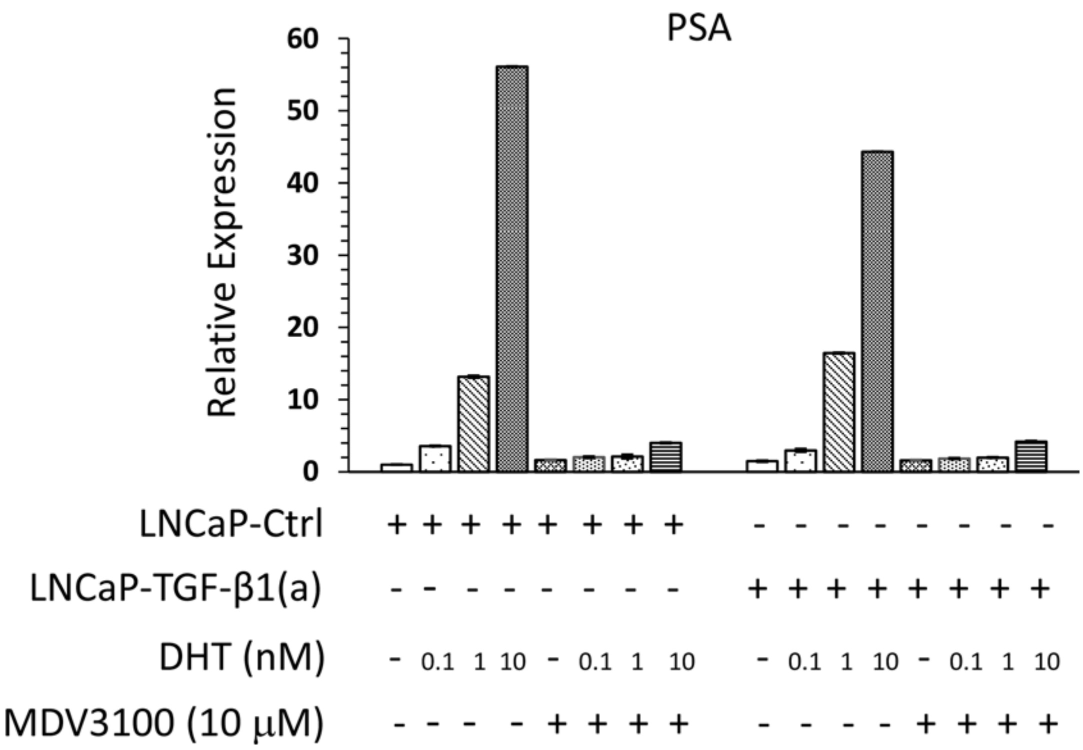

Supplementary Figure 2: LNCaP-TGF-β1(a) cells and LNCaP-Ctrl cells express comparable levels of basal and DHT-induced PSA. LNCaP-TGF-β1(a) cells and LNCaP-Ctrl cells were cultured in RPMI1640 supplemented with 0.2% charcoal-stripped FBS, and treated with different dosages of DHT in the presence (+) or absence (-) of 10 μM of MDV3100 for 6 days. Total RNA was extracted and reverse transcribed. qPCR were used to analyze the expression of PSA in these co-cultures. Representative data is from two independent experiments. All gene expression data were normalized to GAPDH expression.
